# Impact of Age of Onset on Survival after Hepatectomy for Patients with Colorectal Cancer Liver Metastasis: A Real-World Single-Center Experience

**DOI:** 10.3390/curroncol29110666

**Published:** 2022-11-06

**Authors:** Hong-Wei Wang, Li-Jun Wang, Ke-Min Jin, Quan Bao, Juan Li, Si-Kai Ge, Kun Wang, Bao-Cai Xing

**Affiliations:** 1 Hepatopancreatobiliary Surgery Department I, Key Laboratory of Carcinogenesis and Translational Research, Ministry of Education, Peking University School of Oncology, Beijing Cancer Hospital and Institute, Haidian District, Beijing 100142, China; 2 Department of Mathematics Science, Xi’an Jiaotong Liverpool University, Suzhou 215127, China

**Keywords:** colorectal liver metastasis, age of onset, hepatectomy, RAS status

## Abstract

Purpose: The incidence of early-onset CRC is increasing. However, the effect of age of onset on the long-term outcome of colorectal cancer liver metastasis (CRLM) remains unclear. This study aimed to evaluate the association between the age of onset and the oncological outcome of CRLM patients and to investigate whether the prognostic role of RAS mutation is altered with age. Methods: We retrospectively investigated consecutive patients at our institution who underwent initial liver resection between 2006 and 2020. The inverse probability of treatment weighting (IPTW) method was used to balance the confounders among early- (≤45 years; EOCRLM), intermediate- (46–70 years; IOCRLM), and late-onset (>70 years; LOCRLM) groups. The prognostic role of RAS was assessed based on age group. Results: A total of 1189 patients were enrolled: 162 in the EOCRLM group, 930 in the IOCRLM group, and 97 in the LOCRLM group. No difference in disease-free survival (DFS) was found between the three groups. However, EOCRLM were more likely to develop extrahepatic and extrapulmonary metastasis and had significantly lower five-year OS rates than IOCRLM. After IPTW, EOCRLM remained a negative prognostic predictor. RAS mutations were significantly associated with worse survival than wild-type RAS in EOCRLM and IOCRLM. However, RAS mutation did not predict the prognosis of patients with LOCRLM. Conclusions: Patients with EOCRLM had a significantly lower OS than IOCRLM patients and age influences the prognostic power of RAS status. These findings may be helpful for doctors to guide the clinical treatments and develop follow-up strategies.

## 1. Introduction

In China, colorectal cancer (CRC) ranks second among the most common cancers and fifth as the leading cause of cancer death [1]. The number of people with newly diagnosed CRC in China is projected to reach 0.91 million by 2040, a 64% increase from that in 2020 [2]. Although the incidence rates for CRC have stabilized or declined among older adults in some developed countries, the incidence of CRC among younger people has been increasing globally in recent years [3,4,5,6]. Numerous studies have demonstrated that young patients commonly manifest distinctive clinicopathologic features; that is, they have advanced-stage diseases at presentation, predominantly left-sided colon and rectal cancers, and mucinous or signet ring histology, and may harbor fewer KRAS/BRAF mutations [7,8,9].

Up to 50% of CRC patients will eventually develop liver metastasis during their lifetime [10], which is the major cause of death [11]. Comprehensive treatment based mainly on surgery is generally recognized as the best treatment option for patients with colorectal liver metastasis (CRLM). Several factors, including KRAS/BRAF mutation, the size of the largest tumor, the number of hepatic nodules, elevated serum tumor markers (CEA and CA 19-9), the interval between the primary tumor and the diagnosis of metastasis, the location, and the positive lymph node of the primary tumor have been found to be associated with oncological outcome after hepatectomy [12,13,14,15,16]. However, the effect of age on the long-term outcome of CRLM remains unclear. Several retrospective studies have suggested that older people have a poorer prognosis than younger people [17,18], while another study reported that age above 60 years was not associated with poorer long-term survival [19]. In addition, Alexandre et al. found that early-onset CRLM (EOCRLM) demonstrated comparable long-term survival to late-onset CRLM (LOCRLM) and showed that age may affect the prognostic value of RAS mutations [20].

In this study, we aimed to investigate the effect of age on outcome in patients who underwent surgical resection of CRLM and to determine whether RAS mutation shows different prognostic effects among patients of different ages in a large center in China.

## 2. Methods

### 2.1. Study Population

All patients who underwent their first radical liver resection for CRLM between January 2006 and December 2020 at the Hepato-Pancreato-Biliary Surgery Department I, Peking University Cancer Hospital, were included in this study. Patients who received noncurative resection of the primary tumor or hepatic or extrahepatic metastases or had incomplete clinical data were excluded. Patients who were lost to follow up or died within 90 days postoperatively were also excluded. The survival data in this study were reviewed on 30 November 2021. The present study was approved by the institutional review board.

### 2.2. Patient Management

Aside from the conventional physical examinations and laboratory evaluations, all of the patients underwent several imaging studies, such as enhanced chest and abdominopelvic cavity CT and/or abdominal MRI scans, to evaluate the presence of extrahepatic disease and assess resectability for CRLM. Full-length fluorouracil-based perioperative chemotherapy was recommended unless the patient had as many good prognostic factors as possible (such as single, resectable metachronous metastases with long disease-free intervals) or the patient refused. The primary lesion or CRLM was routinely subject to genetic testing for RAS and BRAF mutations after 2014. For patients who underwent surgery prior to 2014, molecular analysis was carried out on archived formalin-fixed and paraffin-embedded tumor tissue specimens.

All surgical procedures were performed by experienced surgeons who performed at least 50 procedures a year and with similar operation techniques in our center. All surgical procedures were performed using standard hepatectomy techniques. First, surgical exploration was performed through a median laparotomy or laparoscopy. Then, intraoperative ultrasound was conducted to determine the number of tumors, confirm the exact tumor location, and search for lesions not seen on preoperative imaging. Hepatic parenchymal transection was performed using a harmonic scalpel (Ethicon Endo-Surgery, Cincinnati, OH, USA) and/or a Peng multifunction operative dissector. The Pringle maneuver was applied intermittently according to the surgeon’s preference. Postoperative care was conducted according to standard postoperative protocols at our center.

A follow-up evaluation was conducted every three months during the first two years, every six months during the next three years, and once a year thereafter for all patients. The follow-up items included physical examinations, tumor marker measurements, liver function tests, radiological imaging with enhanced abdominal MRI scans or computed tomography, and chest and pelvic computed tomography scans.

### 2.3. Data Collection

The following data on patient demographics, cancer-specific clinicopathological variables, and surgery-related characteristics were collected: age, sex, BMI before hepatectomy, primary tumor characteristics (primary tumor location [primary tumors located in the cecum to transverse colon were recorded as right sided, whereas tumors located from the splenic flexure to rectum were considered left sided], T stage [tumor depth], N stage [lymph node invasion]), use of preoperative chemotherapy, CRLM characteristics (prehepatectomy CEA level, type of CRLM [metachronous or synchronous, synchronous disease was defined as a diagnosis of CRLM at or before primary colorectal tumor diagnosis], the largest tumor size, number of nodules, metastatic distribution, and RAS/BRAF status), operative variables (intraoperative blood transfusion, extent of hepatectomy [minor or major resection]), use of other local therapy including radiofrequency ablation or stereotactic body radiation therapy, resection margin status [R0 or R1), presence of extrahepatic disease (EHD), use of adjuvant treatments, and patterns of recurrence (intrahepatic recurrence, pulmonary metastasis, or other metastatic sites).

### 2.4. Definitions and Grouping

EOCRLM is defined as CRLM diagnosed before the age of 45, as the newly updated guidelines recommend that CRC screening begins at the age of 45 years [21]. We also defined LOCRLM (>70 years) and intermediate-onset (IOCRLM; between 45 and 70 years) groups of patients at the time of diagnosis. Overall survival (OS) was measured as the time from the date of surgery to either death or the date of last follow up. Disease-free survival (DFS) was defined as the time interval between the day of surgery and the day of cancer recurrence. Resection margins were defined as R1 if the tumor had microscopic involvement or involvement within 1 mm of the margins. Major hepatectomy was defined as resection of three or more liver segments according to the Couinaud classification, whereas minor hepatectomy was comprised partial hepatectomy of less than three segments.

### 2.5. Statistical Analysis

The primary end point of this study was OS. The secondary endpoints were the value of prognostic factors at different ages. The normality distribution of variables was evaluated by using the Kolmogorov–Smirnov and Shapiro–Wilk tests. Parametric continuous data are expressed as the mean ± SD, and the median and interquartile range (IQR) are used to describe the nonparametric data. Categorical data are expressed as numbers and percentages. Categorical variables are expressed as absolute numbers with related percentages (*n*, %). Demographic, cancer-specific clinicopathological and surgery-related characteristics were compared using Fisher’s exact test, the chi square test, or ANOVA as appropriate. OS analysis was performed using Kaplan–Meier curves and the log-rank test in the univariable analyses and restricted cubic spline (RCS) curves (continuous age variable). Multivariate regression analyses were performed to further examine the effects of the interaction between age (continuous age variable) and RAS status. Age-specific mortality rates were estimated by the number of patients who died/the total patient numbers in different age groups who had different RAS statuses. Multivariable analyses were conducted on significant variables identified in the univariable analyses using Cox proportional hazards models (categorical age variables).

To minimize the imbalance of possible confounders among age categories, inverse probability of treatment weighting (IPTW) was performed to adjust for confounding due to differences between the three groups, assigning a weight of mean of propensity scores (PS) for the IOCRLM group and (1 − means of PS)/(1 − PS) for the other two groups, where PS is the probability that each individual will be assigned to the EOCRLM group. In our model, 14 covariates (sex, primary tumor location, N stage, Ras status, bilobular disease, largest tumor size, number of nodules, extrahepatic disease, CEA level, major hepatectomy, intraoperative transfusion, R1 resection, and received preoperative or adjuvant chemotherapy) were selected to perform IPTW. After IPTW, three generated populations of different sizes from the original dataset were obtained, and the characteristics were comparable between the groups. All statistical analyses were computed using IBM SPSS Statistics 23.0 (IBM Corp., Armonk, NY, USA) and the open-source R Studio version 1.2.5033 (Posit software, Boston, MA, USA). dplyr, RISCA, survminer, WeightIt, ipw, coin, Visreg, yardstick packages were used). A *p* value < 0.05 was considered significant.

## 3. Results

### 3.1. Patients and Characteristics

Between 2006 and 2020, 1331 patients underwent 1483 liver resections for CRLM. Of these patients, a total of 142 cases were excluded from the survival analysis (lost to follow up in 44 cases, 90-day postoperative death in 4 cases, incomplete resection in 43 patients, incomplete data in 19 patients, and undergoing repeat hepatectomy only in 32 patients). The median age for all patients was 58 years (range, 19–83 years). A total of 162 (13.5%) patients had EOCRLM, 930 (78.2%) had IOCRLM, and 97 (8.2%) patients had LOCRLM. Patients with EOCRLM showed a higher prevalence of bilobular disease (*p* = 0.003) and had more tumors (*p* = 0.001) than the IOCRLM and LOCRLM groups. Patients with LOCRLM had larger tumors than the EOCRLM and IOCRLM groups, and fewer LOCRLM patients received preoperative (*p* = 0.001) or adjuvant chemotherapy (*p* = 0.006). There was no significant difference in sex, number of patients with RAS or BRAF mutations, and the frequency of right-sided primary tumors among the different age groups (Table 1).

### 3.2. Relationship of Age with OS

**Figure 1:**
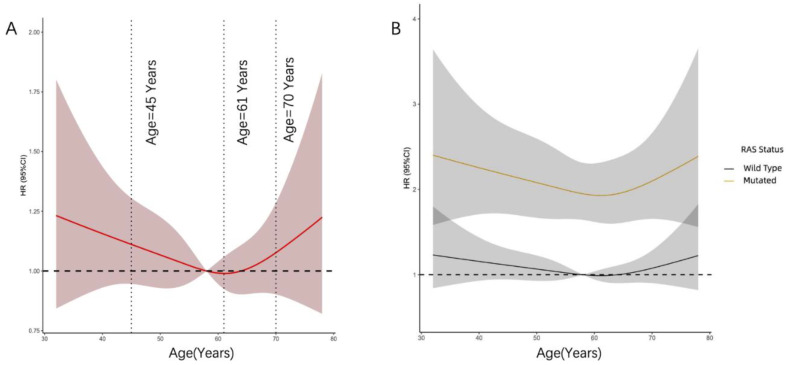
(**A**) The relationship between OS and age in the whole cohort was explored with the RCS function based on the Cox’s equation. (**B**) The effect of the age on the prognosis of CRLM between different RAS status.

The association between age at diagnosis on a continuous scale and OS was depicted as RCS curves adjusted by the Cox model. The curve was U shaped; the young and old were associated with an increased risk of mortality. This association was also found in patients with or without RAS mutations (Figure 1). Based on the above results, patients were divided into three groups for subsequent analyses according to their age.

### 3.3. Survival Analysis

**Figure 2:**
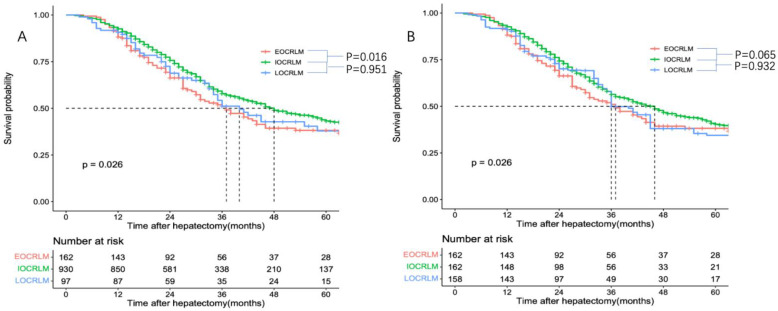
Overall survival among the different age groups before (**A**) and after (**B**) the IPTW.

The median follow up was 28 months (range, 3–188 months) for all patients and 32 months (range, 11–188 months) for survivors. The five-year DFS rates in patients with EOCRLM, IOCRLM, and LOCRLM were 20.5%, 24.7%, and 19.5%, respectively, and the five-year OS rates after hepatectomy for CRLM were 36.7%, 43.2%, and 35% for EOCRLM, IOCRLM, and LOCRLM, respectively. The OS rates were significantly lower in the EOCRLM group than in the IOCRLM group (*p* = 0.016, Figure 2A). No difference in DFS was found between the three groups. (*p* = 0.46, Figure S1). Factors significantly associated with poor OS in the multivariable Cox model were EOCRLM, positive lymph nodes in the primary tumor, RAS mutation, multiple metastases, a tumor size larger than 5 cm, CEA > 20, extrahepatic metastasis, major resection, positive resection margin, and no adjuvant chemotherapy (Table S1).

Due to the potential difference among the three groups, IPTW was performed to weight the imbalanced characteristics (sex, primary tumor location, N stage, Ras status, bilobular disease, largest tumor size, number of nodules, extrahepatic disease, CEA level, major hepatectomy, intraoperative transfusion, R1 resection, and received preoperative or adjuvant chemotherapy), and three new comparable cohorts were created. The characteristics of the weighted cohorts are shown in Table S2. The median survival time in the IOCRLM cohort (46 months) was also approaching significant longer than the median survival time in the EOCRLM cohort (37 months) (*p* = 0.065 Figure 2B). In addition, multivariate analysis suggested that IOCRLM (*p* = 0.014), positive lymph nodes in the primary (*p* = 0.045), RAS mutation (*p* = 0.000), a tumor size larger than 5 cm (*p* = 0.025), CEA > 20 (*p* = 0.014), extrahepatic metastasis (*p* = 0.016), and a positive resection margin (*p* = 0.010) were independent predictors of OS (Table 2).

### 3.4. The Impact of RAS Mutations on Survival in Different Age Groups

The median survival time was significantly shorter among patients with RAS mutations than among patients without mutations (32 months vs. 62 months, *p* < 0.001) in the whole cohort. However, though no interaction was found between age and RAS in the multivariate analysis (*p* = 0.164 Table S1), our study shows that RAS mutation has no effect on the prognosis of older patients according to age-specific mortality analysis (Figure S2). To further identify the effect of RAS mutations on prognosis in different age groups, we conducted subgroup analyses. The patients with mutant RAS had shorter OS than those with wild-type RAS in the EOCRLM (63 vs. 27 months, *p* = 0.001) and IOCRLM (60 vs. 33 months, *p* < 0.001) groups (Figure S3A,B). The difference in survival between patients with mutant and wild-type RAS in the LOCRLM group (58 vs. 34 months, *p* = 0.095) was statistically nonsignificant (Figure S3C, Table S3). Similar negative effects of RAS mutation on OS were observed in the EOCRLM (HR: 1.95 [95% CI: 1.26–3.04], *p* = 0.003) and IOCRLM (HR: 1.90 [95% CI: 1.55–2.33], *p* < 0.001) groups after multivariable Cox regression analysis (Tables S4 and S5).

### 3.5. Recurrence Patterns and Subsequent Therapy in Different Age Groups

Overall, 845 patients (71.1%) experienced recurrence. The most common site of recurrence was the liver (*n* = 655, 55.1%), followed by the lungs (*n* = 303, 25.5%), lymph nodes (*n* = 112, 9.4%), bone (*n* = 54, 4.5%), and peritoneum (*n* = 52, 4.4%). The proportion of patients with hepatic recurrence and pulmonary metastases was similar among the three age groups. Patients with EOCRLM experienced a significantly higher proportion of extrahepatic and extrapulmonary metastasis than patients in the other two groups. Patients who suffered recurrence in the LOCRLM group were less likely to receive salvage resection or local therapy than those patients in the other groups (Table 3).

## 4. Discussion

In this study, we conducted a detailed retrospective analysis to assess the differences in prognosis, recurrence patterns, and influence of RAS mutation on prognosis among EOCRLM, IOCRLM, and LOCRLM patients. Our results show that no difference in DFS was found between age groups, while IOCRLM patients had superior OS compared with other groups. The recurrence patterns and the prognostic significance of RAS mutations varied between different groups. Patients with EOCRLM develop extrahepatic and extrapulmonary metastasis more frequently, and RAS mutations did not affect the long-term outcome of the LOCRLM population.

Early-onset CRC has been reported to have distinctive pathological and molecular characteristics [7,8,9,10]. However, there are just a few articles describing the clinical, pathological, and molecular characteristics of patients with EOCRLM, IOCRLM, and LOCRLM. Our study did not support the notion that younger patients were more likely to have tumors with more aggressive histologic subtypes. In this study, EOCRLM was not associated with poor tumor differentiation or mucinous or signet ring adenocarcinoma. The three groups had similar proportions of patients with left-sided primary CRC, synchronous metastasis, and RAS/BRAF mutations. Meanwhile, our research revealed that patients with EOCRLM had multiple tumors and bilateral metastasis more frequently, while patients with LOCRLM had larger tumor diameters. Older patients have been reported to receive less intensive treatment than younger patients [22,23]. Our results are consistent with those studies. The EOCRLM and IOCRLM groups underwent major hepatectomy more often and were more likely to be treated with perioperative chemotherapy than the LOCRLM group.

Surgery remains the cornerstone of potentially curative treatment for CRLM. In light of the prognostic effect of age after hepatectomy, our results were in conflict with previous studies [19,20]. Our study showed that the prognosis of early-onset patients with CRLM is worse than that of IOCRLM. In previous studies, all older patients were merged into a single group, and these studies did not take into consideration prognostic distinctions among IOCRLM and LOCRLM. It has been suggested that intermediate-onset CRC behaves like the transitional group between early- and late-onset CRC and exhibits several unique clinicopathological and molecular characteristics [24,25]. Hence, we hypothesized that the overall prognosis from this group of patients may also behave as a translational one and may differ compared with the other patients with CRLM. Our conjecture has been confirmed by prognostic analysis. As shown in our study, a substantial reduction in the risk of mortality was seen in the RCS plot, which reached the lowest risk at approximately 61 years and then increased thereafter.

To attempt to minimize significant baseline differences in clinical variables between patient cohorts, IPTW analysis was then introduced. In pairwise comparisons, compared with IOCRLM, EOCRLM still had significantly shorter survival in multivariable analysis. Our results showed that age was related to the OS without statistically significant influence on DFS. A possible explanation for the worse outcomes in EOCRLM in our study is that younger individuals were more prone to develop extrahepatic and extrapulmonary lesions after hepatectomy. This finding is in line with a recent study focused on CRC patients, in which under age 50 years at diagnosis was an independent risk factor for multiorgan metastasis [26]. Another reason that may explain our finding is related to the difference in molecular features between groups. Younger CRC patients often have fewer APC mutations and present with consensus molecular subtype 1 [5,27], which has been associated with poor OS [27,28]. As EOCRLM demonstrated a worse prognosis and had a superior performance status compared with the remaining study population. Thus, these patients may need more extensive perioperative therapy and more comprehensive follow-up after surgery.

Numerous studies have shown that RAS mutation is associated with poorer survival following curative resection of CRLM [14,29,30]. However, some studies have recently suggested that RAS mutations have different roles when predicting prognosis in CRLM with distinct clinical characteristics [20,31]. Similar to previous reports, our study also showed that RAS mutation did not have the same prognostic effect among patients of different ages [20,32]. In the current study, RAS mutation was found to be a prognostic marker for survival among patients with EOCRLM and IOCRLM, but was not found to be a prognostic factor among patients with LOCRLM. One reason for this discrepancy is probably due to the different treatment choices. The LOCRLM group in this study was less likely to be treated with perioperative chemotherapy. Takeda et al. reported that the difference in prognostic value of RAS mutation seems to be associated with the use of preoperative chemotherapy. When the proportion of patients receiving preoperative chemotherapy was less than 30%, RAS mutation was not a prognostic factor of poor clinical outcome [31]. Surgical resection and local therapy are primary options for treating tumor recurrence after hepatectomy and are associated with significantly longer post-recurrence survival. Patients with RAS mutations were typically less fit than those with wild-type RAS to receive surgery or local therapy after recurrence [33], which could partially explain the survival differences between the RAS mutation and wild-type groups. In the present study, only a minority of LOCRLM patients underwent salvage resection or local therapy after recurrence, which may potentially produce compensatory effects for biological differences. However, there may be several other causes for the difference. Therefore, further research needs to be performed to investigate the reason.

Primary tumor location has been identified as a prognostic factor in patients with advanced metastatic CRC [34]. For resectable CRLM, two recent meta-analyses demonstrated that a left-sided primary tumor is a substantially better prognostic factor in terms of OS [35,36]. However, the result of the present study varied from that of these meta-analyses. In our study, primary tumor location has no influence on survival after hepatic resection for CRLM. CRLM is a complex and heterogeneous disease. Although the two meta-analyses found a prognostic role for PTL in terms of OS, approximately half of the enrolled studies [5/12, 22/43] did not show a better OS for left-sided CRLM, making the results paradoxical. Furthermore, a recent study by the International Genetic Consortium for CRLM concluded that right sided is a good predictor of overall survival (OS) only in patients with K-RAS wild-type tumors [13]. In the future, the impact of the primary site on the OS of resectable CRLM should be further investigated by well-designed prospective studies.

We acknowledge that this study contains some limitations that should be noted when generalizing the conclusions. First, although IPTW and multivariable analysis were performed, the single institution and explorative and descriptive retrospective nature of the study still limits the extrapolation of the results. Second, microsatellite instability status was not evaluated because less than forty percent of patients underwent this type of examination in this study. Thus, we were unable to determine whether the patients had Lynch syndrome. Third, other potential confounding factors of prognosis, such as socioeconomic status, history of inflammatory bowel disease, and specific reasons for therapy failure after recurrence, were not listed and analyzed due to a lack of available data. Fourth, the current study is limited by its long timeframe. Though preoperative and adjuvant therapy were taken into account in the IPTW, treatments were not standardized given the long study period, which leads to systematic bias. Fifth, this study did not detect more mutations (such as TP53, SMAD family) due to its retrospective nature. Several studies have confirmed the role of these genes in predicting prognosis [37]. Last, all the patients enrolled in this study were of Asian ethnicity from China. To confirm our results, further studies including external validation should be conducted.

In conclusion, the present study indicates that patients with EOCRLM were more likely to have bilobular disease, had more tumors, and developed extrahepatic and extrapulmonary metastasis more frequently. EOCRLM was associated with significantly worse OS than IOCRLM, and the prognostic impact of RAS mutation diminished with advancing age. The exact reason for this is still unclear. More high-quality studies are needed to address this issue in the future.

## Figures and Tables

**Table 1:** Patient characteristics according to age group before IPTW.

Variables	EOCRLM (162)	IOCRLM (*n* = 930)	LOCRLM (*n* = 97)	*p*
Patient characteristics				
Age (i.q.r), years	41 (37.0–43.0)	59.0 (53.0–63.0)	73 (71.0–76.0)	0.000
Sex				
Male (%)	103 (63.6)	607 (65.3)	65 (67.0)	0.848
Primary tumor characteristics				
Right-sided tumor (%)	25 (15.4)	158 (17.0)	24 (24.7)	0.124
Poor tumor differentiation (%)	29 (17.9)	147 (15.8)	19 (19.6)	0.542
Mucinous or signet cell (%)	5 (3.1)	37 (3.9)	1 (1.0)	0.218
T stage				
T3 or T4 stage (%)	150 (92.6)	838 (90.1)	90 (92.8)	0.457
Node-positive primary tumor	121 (74.7)	654 (70.3)	63 (64.9)	0.244
Preoperative factors				
Preoperative chemotherapy (%)	133 (82.1)	721 (77.5)	60 (61.9)	0.001
Preoperative CEA > 20 (%)	43 (26.5)	254 (27.3)	30 (30.9)	0.718
Synchronous CLM	108 (66.7)	570 (61.3)	53 (54.6)	0.152
CRLM characteristics				
Tumor number (i.q.r), cm	3 (1–6)	2 (1–5)	2 (1–4)	0.001
Tumor number (Multiple)	119 (73.5)	617 (66.3)	55 (56.7)	0.021
Maximum tumor size (i.q.r), cm	2.4 (1.5–3.5)	2.5 (1.6–3.8)	3 (2.1–4.5)	0.002
Maximum tumor size ≥ 5 cm (%)	59 (36.4)	406 (43.7)	54 (55.7)	0.010
Bilateral disease (%)	100 (61.7)	477 (51.3)	39 (40.2)	0.003
Ras mutation (%)	65 (40.1)	358 (38.5)	438 (44.3)	0.516
Braf mutation (%)	5 (3.1)	11 (1.2)	1 (1.0)	0.160
Extrahepatic metastasis (%)	36 (22.1)	151 (16.2)	15 (15.5)	0.159
Hepatic resection				
Plus ablation (%)	39 (24.1)	202 (21.7)	13 (13.4)	0.109
Major hepatectomy (%)	43 (26.5)	207 (22.3)	12 (12.4)	0.027
Blood loss (i.q.r), mL	200 (100–300)	200 (100–250)	100 (100–200)	0.148
Intraoperative transfusion (%)	17 (10.5)	57 (6.1)	9 (9.3)	0.086
R1 resection	33 (20.3)	198 (21.3)	17 (17.5)	0.677
Adjuvant chemotherapy	123 (75.9)	692 (74.4)	58 (59.8)	0.006

**Table 2:** Univariate and multivariable Cox regression analysis of overall survival in the entire cohort after IPTW.

	Univariate		Multivariate	
	HR (95% CI)	*p*	HR (95% CI)	*p*
Age group				
EOCRLM	Ref		Ref	
IOCRLM	0.79 (0.63–1.01)	0.065	0.73 (0.57–0.94)	0.014
LOCRLM	0.98 (0.66–1.46)	0.932		
Female gender	0.81 (0.68–0.96)	0.017	0.79 (0.59–1.07)	0.132
Primary tumor location				
Left-sided primary	Ref			
Right-sided primary	1.33 (1.05–1.67)	0.007	0.90 (0.61–1.34)	0.615
Primary tumor stage				
T1 & T2	Ref			
T3 & T4	1.17 (0.89–1.57)	0.288	–	
Lymph node metastasis	1.39 (1.16–1.67)	0.001	1.46 (1.01–2.12)	0.046
Preoperative chemotherapy	1.10 (0.90–1.33)	0.349		
CEA > 20 ng/dL	1.73 (1.42–2.11)	0.000	1.47 (1.08–1.99)	0.014
Synchronous liver metastases	1.09 (0.92–1.30.)	0.293	–	
>1 liver metastasis	1.42 (1.19–1.67)	0.000	1.32 (0.88–1.99)	0.180
Maximum tumor size ≥ 5 cm	1.54 (1.30–1.83)	0.000	1.36 (1.04–1.78)	0.025
Bilateral liver disease	1.34 (1.13–1.59)	0.000	1.34 (0.92–1.94)	0.131
RAS stasus				
Wild-type tumors	Ref		Ref	
Mutated	1.93 (1.61–2.31)	0.000	2.02 (1.51–2.70)	0.000
BRAF status				
Wild-type tumors	Ref			
Mutated	1.04 (0.38–2.84)	0.936		
Extrahepatic disease	1.85 (1.44–2.39)	0.000	1.50 (1.08–2.08)	0.016
Major resection	1.49 (1.20–1.86)	0.000	1.29 (0.86–1.92)	0.230
Red blood cell transfusion	1.43 (1.03–2.00)	0.010	1.23 (0.77–1.98)	0.385
R1 resection	1.29 (1.05–1.58)	0.009	1.49 (1.10–1.51)	0.010
Adjuvant chemotherapy	0.67 (0.59–0.77)	0.000	0.81 (0.60–1.09)	0.173

**Table 3:** Recurrence patterns and subsequent therapy in CRLM patients with different age groups.

Variables	EOCRLM (162)	IOCRLM (930)	LOCRLM (97)	*p*
Intrahepatic recurrence (%)	89 (54.9)	506 (54.4)	60 (61.9)	0.373
Pulmonary metastasis (%)	40 (24.7)	242 (26.0)	21 (21.6)	0.623
Other sites (%)	53 (32.7)	202 (21.7)	18 (18.6)	0.005
Salvage resection (%)	22 (22.0)	115 (20.7)	4 (6.6)	0.025
Local therapy	49 (49.0)	299 (53.7)	22 (36.1)	0.797

## Data Availability

The data presented in this study are available on request from the corresponding author.

## Supplementary Materials

The following supporting information can be downloaded at: https://www.mdpi.com/article/10.3390/curroncol29110666/s1, Figure S1: Kaplan–Meier estimates of disease-free survival stratified by age groups in the whole cohort. Figure S2: The age-specific mortality risk by RAS status. Figure S3: Survival analysis according to the risk stratification a, b and c: Kaplan–Meier curves of the EOCRLM group patients(a), IOCRLM group(b) and LOCRLM group(c) stratified by the RAS status. Table S1: Univariate and multivariable Cox regression analysis of overall survival in the entire cohort. Table S2: Patient characteristics according to age group before and after IPTW. Table S3: Univariate and multivariable Cox regression analysis of overall survival in the LOCRLM cohort. Table S4: Univariate and multivariable Cox regression analysis of overall survival in the EOCRLM cohort. Table S5: Univariate and multivariable Cox regression analysis of overall survival in the IOCRLM cohort
