# Supplementary material for: Impact of Age of Onset on Survival after Hepatectomy for Patients with Colorectal Cancer Liver Metastasis: A Real-World Single-Center Experience
Source: Curr Oncol. 2022 Nov 6;29(11):8456–67. doi: 10.3390/curroncol29110666 (PMC9689561; doi:10.3390/curroncol29110666)
Supplement: Supplementary file 1 [file curroncol-29-00666-s001.zip › curroncol-1939929-supplementary.pdf]

Current Oncology

Supplementary Material

Impact of Age of Onset on Survival after Hepatectomy for Patients with Colorectal Cancer Liver Metastasis: A Real-World Single-Center Experience

Hong-Wei Wang, Li-Jun Wang, Ke-Min Jin, Quan Bao, Juan Li, Si-Kai Ge, Kun Wang and Bao-Cai Xing

Table S1. Univariate and multivariable Cox regression analysis of overall survival in the entire cohort.

	**Univariate**		**Multivariate**	
	**HR (95% CI)**	***p***	**HR (95% CI)**	***p***
Age group				
EOCRLM	Ref			
IOCRLM	0.75(0.60-0.95)	0.016	0.78 (0.60-0.96)	0.019
LOCRLM	1.05(0.67-1.34)	0.951		
Female gender	0.81 (0.68-0.96)	0.017	0.87 (0.73-1.05)	0.140
Primary tumor location				
Left-sided primary	Ref			
Right-sided primary	1.33 (1.08-1.64)	0.001	1.13 (0.91-1.40)	0.273
Primary tumor stage				
T1 & T2	Ref			
T3 &T4	1.18 (0.87-1.60)	0.290	-	
Lymph Node metastasis	1.40 (1.15-1.69)	0.000	1.32 (1.08-1.60)	0.006
Preoperative chemotherapy	1.10 (0.90-1.34)	0.351		
CEA >20 ng/dl	1.75 (1.46-2.08)	0.000	1.55 (1.29-1.86)	0.000
Synchronous liver metastases	1.10 (0.92-1.30.)	0.293	-	
>1 liver metastasis	1.43 (1.18-1.72)	0.000	1.35 (1.06-1.72)	0.016
Maximum tumor size ≥ 5cm	1.55 (1.31-1.63)	0.000	1.42 (1.19-1.70)	0.000
Bilateral liver disease	1.35 (1.14-1.60)	0.000	1.08 (0.87-1.35)	0.471
RAS stasus				
Wild-type tumors	Ref		Ref	
Mutated	1.95 (1.65-2.32)	0.000	1.88 (1.58-2.24)	0.000
BRAF status				
Wild-type tumors	Ref			
Mutated	1.04(0.39-2.78)	0.941		
Extrahepatic disease	1.88 (1.53-2.32)	0.000	1.84 (1.49-2.27)	0.000
Major resection	1.50 (1.23-1.82)	0.000	1.29 (1.05-1.58)	0.014
Red Blood Cell Transfusion	1.44 (1.09-1.92)	0.011	1.11 (0.83-1.50)	0.414
R1 resection	1.29 (1.07-1.57)	0.009	1.31 (1.08-1.60)	0.007
Adjuvant chemotherapy	0.67 (0.56-0.80)	0.000	0.71 (0.59-0.85)	0.000
Interaction between age and RAS status	1.51 (1.32-1.73)	0.000	0.84 (0.65-1.08)	0.164

CEA, carcinoembryonic antigen; T CI, confidence interval.

Table S2. Patient characteristics according to age group before and after IPTW.

		Before IPTW				After IPTW			
**Age Group**		**EOCRLM**	**AOCRLM**	**LOCRLM**	***p***	**EOCRLM**	**AOCRLM**	**LOCRLM**	***p***
**n**		162	930	97		162	162	158	
**gender**	female	59 (36.4)	323 (34.7)	32 (33.0)	0.848	59 (36.4)	59 (36.5)	56 (35.5)	0.959
	male	103(63.6)	607 (65.3)	65 (67.0)		103 (63.6)	103 (63.5)	102 (64.5)	
**Primary tumor location**	Left-sided	137 (84.6)	772 (83.0)	73 (75.3)	0.124	137 (84.6)	137 (84.6)	133 (84.3)	0.992
	Right-sided	25 (15.4)	158 (17.0)	24 (24.7)		25 (15.4)	25 (15.4)	25 (15.7)	
**T stage**	T1/T2	12 (7.4)	92 (9.9)	7 (7.2)	0.457	12 (7.4)	15 (9.3)	15 (9.5)	0.762
	T3/T4	150 (92.6)	838 (90.1)	90 (92.8)		150 (92.6)	147 (90.7)	143 (90.5)	
**Lymph Node metastasis**	No	41 (25.3)	276 (29.7)	34 (35.1)	0.244	41 (25.3)	41 (25.3)	47 (29.7)	0.579
	Yes	121 (74.7)	654 (70.3)	63 (64.9)		121 (74.7)	121 (74.7)	111 (70.3)	
**RAS status (%)**	Wild-type	97 (59.9)	572 (61.5)	54 (55.7)	0.516	97 (59.9)	96 (59.5)	104 (65.7)	0.494
	mutated	65 (40.1)	358 (38.5)	43 (44.3)		65 (40.1)	66 (40.5)	54 (34.3)	
**BRAF status (%)**	Wild-type	157 (96.9)	919 (98.8)	96 (99.0)	0.160	157 (96.9)	160 (98.9)	157 (99.4)	0.177
	mutated	5 (3.1)	11 (1.2)	1 (1.0)		5 (3.1)	2 (1.1)	1 (0.6)	
**Preoperative chemotherapy**	No	29 (17.9)	209 (22.5)	37 (38.1)	0.001	29 (17.9)	29 (17.9)	31 (19.6)	0.824
	Yes	133 (82.1)	721 (77.5)	60 (61.9)	0.001	133 (82.1)	133 (82.1)	127 (80.4)	0.824
**Synchronous metastases**	No	54 (33.3)	360 (38.7)	44 (45.4)	0.152	54 (33.3)	56 (34.6)	73 (46.3)	0.101
	Yes	108 (66.7)	570 (61.3)	53 (54.6)		108 (66.7)	106 (65.4)	85 (53.7)	
**Bilateral liver disease**	No	62 (38.3)	453 (48.7)	58 (59.8)	0.003	62 (38.3)	62 (38.3)	58 (36.7)	0.896
	Yes	100 (61.7)	477 (51.3)	39 (40.2)		100 (61.7)	100 (61.7)	100 (63.3)	
**R1 resection**	No	129 (79.6)	732 (78.7)	80 (82.5)	0.677	129 (79.6)	129 (79.6)	130 (82.3)	0.798
	Yes	33 (20.4)	198 (21.3)	17 (17.5)		33 (20.4)	33 (20.4)	28 (17.7)	
**Red blood cell Transfusion**	No	145 (89.5)	873 (93.9)	88 (90.7)	0.086	145 (89.5)	145 (89.5)	145 (91.8)	0.737
	Yes	17 (10.5)	57 (6.1)	9 (9.3)		17 (10.5)	17 (10.6)	13 (8.4)	
**>1 liver metastasis**	No	43 (26.5)	313 (33.7)	42 (43.3)	0.021	39 (24.1)	39 (24.1)	34 (21.5)	0.763
	Yes	119 (73.5)	617 (66.3)	55 (56.7)		123 (75.9)	123 (75.9)	124 (78.5)	
**Extrahepatic disease**	No	126 (77.8)	779 (83.8)	82 (84.5)	0.159	119 (73.5)	119 (73.5)	120 (76.0)	0.799
	Yes	36 (22.2)	151 (16.2)	15 (15.5)		43 (26.5)	43 (26.5)	38 (24.0)	
**Maximum tumor size ≥ 5cm**	No	103 (63.6)	524 (56.3)	43 (44.3)	0.010	126 (77.8)	125 (77.4)	132 (83.5)	0.433
	Yes	59 (36.4)	406 (43.7)	54 (55.7)		36 (22.2)	37 (22.6)	26 (16.5)	
**CEA >20 ng/dl**	No	119 (73.5)	676 (72.7)	67 (69.1)	0.718	103 (63.6)	103 (63.6)	106 (67.1)	0.744
	Yes	43 (26.5)	254 (27.3)	30 (30.9)		59 (36.4)	59 (36.4)	52 (32.9)	
**Adjuvant chemotherapy**	No	39 (24.1)	238 (25.6)	39 (40.2)	0.006	43 (26.5)	42 (26.0)	44 (27.8)	0.921
	Yes	123 (75.9)	692 (74.4)	58 (59.8)		119(73.5)	120 (74.0)	114 (72.2)	
**Major resection**	No	119 (73.5)	723 (77.7)	85 (87.6)	0.027	123 (75.9)	123 (75.9)	124(78.5)	0.763
	Yes	43 (26.5)	207 (22.3)	12 (12.4)		39 (24.1)	39 (24.1)	34 (21.5)	

CEA, carcinoembryonic antigen.

Table S3. Univariate and multivariable Cox regression analysis of overall survival in the LOCRLM cohort.

	Univariate		Multivariate	
	**HR (95% CI)**	***p***	**HR (95% CI)**	***p***
Female gender	0.97 (0.54-1.74)	0.909		
Primary tumor location				
Left-sided primary	Ref			
Right-sided primary	1.14 (0.60-2.19)	0.694		
Primary tumor stage				
T1 & T2	Ref			
T3 &T4	1.83 (0.74-4.51)	0.849	-	
Lymph Node metastasis	1.81 (0.97-3.35)	0.061	1.75 (0.93-3.31)	0.084
Preoperative chemotherapy	1.30 (0.74-2.29)	0.360		
CEA >20 ng/dl	1.93 (1.11-3.39)	0.021	1.78 (0.99-3.17)	0.054
Synchronous liver metastases	1.37 (0.79-2.39)	0.268	-	
>1 liver metastasis	1.58 (0.90-2.77)	0.110		
Maximum tumor size ≥ 5cm	1.70 (1.39-2.07)	0.083	1.56 (0.84-2.92)	0.159
Bilateral liver disease	2.04 (1.16-3.60)	0.014	2.49 (1.35-4.59)	0.003
RAS stasus				
Wild-type tumors	Ref		Ref	
Mutated	1.58 (0.92-2.74)	0.098	1.38 (0.77-2.46)	0.276
BRAF status				
Wild-type tumors	Ref			
Mutated	1.00(0.00-267)	1.000		
Extrahepatic disease	1.42 (0.68-2.95)	0.346		
Major resection	1.09 (0.49-2.44)	0.838		
Red Blood Cell Transfusion	2.01 (0.85-4.75)	0.113		
R1 resection	1.64 (0.86-3.14)	0.136		
Adjuvant chemotherapy	1.07 (0.61-1.87)	0.820		

Table S4. Univariate and multivariable Cox regression analysis of overall survival in the EOCRLM cohort.

	Univariate		Multivariate	
	**HR (95% CI)**	***p***	**HR (95% CI)**	***p***
Female gender	0.73 (0.48-1.12)	0.148		
Primary tumor location				
Left-sided primary	Ref			
Right-sided primary	1.20 (0.69-2.10)	0.516		
Primary tumor stage				
T1 & T2	Ref			
T3 &T4	1.83 (0.74-4.51)	0.191	-	
Lymph Node metastasis	1.98 (1.17-3.34)	0.011	2.00 (1.15-3.47)	0.013
Preoperative chemotherapy	1.16 (0.69-1.95)	0.585		
CEA >20 ng/dl	1.53 (0.97-2.43)	0.070	1.45 (0.90-2.33)	0.124
Synchronous liver metastases	1.10 (0.92-1.30.)	0.202	-	
>1 liver metastasis	1.77 (1.05-2.99)	0.031	1.40 (0.75-2.63)	0.289
Maximum tumor size ≥ 5cm	0.98 (0.64-1.52)	0.940		
Bilateral liver disease	2.03 (1.28-3.22)	0.003	1.49 (0.85-2.59)	0.163
RAS stasus				
Wild-type tumors	Ref		Ref	
Mutated	2.00 (1.30-3.06)	0.001	1.95 (1.26-3.04)	0.003
BRAF status				

Wild-type tumors	Ref			
Mutated	0.05(0.00-73.5)	0.417		
Extrahepatic disease	1.66 (1.03-2.69)	0.038	1.86 (1.13 -3.08)	0.015
Major resection	1.06 (0.66-1.72)	0.807		
Red Blood Cell Transfusion	1.31 (0.69-2.46)	0.408		
R1 resection	1.43 (0.88-2.32)	0.147		
Adjuvant chemotherapy	0.62 (0.39-0.99)	0.043	0.71 (0.44-1.14)	0.015

Table S5. Univariate and multivariable Cox regression analysis of overall survival in the iOCRLM cohort.

	**Univariate**		**Multivariate**	
	**HR (95% CI)**	***p***	**HR (95% CI)**	***p***
Female gender	0.80 (0.66-0.98)	0.034	0.84 (0.68-1.03)	0.098
Primary tumor location				
Left-sided primary	Ref			
Right-sided primary	1.41 (1.10-1.79)	0.006	1.16 (0.91-1.50)	0.235
Primary tumor stage				
T1 & T2	Ref			
T3 &T4	1.10 (0.78-1.54)	0.605	-	
Lymph Node metastasis	1.26 (1.01-1.58)	0.038	1.11 (0.88-1.39)	0.379
Preoperative chemotherapy	1.08 (0.85-1.36)	0.534		
CEA >20 ng/dl	1.77 (1.44-2.17)	0.000	1.50 (1.21-1.85)	0.000
Synchronous liver metastases	1.02 (0.83-1.25)	0.856	-	
>1 liver metastasis	1.36 (1.10-1.68)	0.005	1.34 (1.00-1.78)	0.047
Maximum tumor size ≥ 5cm	1.70 (1.39-2.07)	0.000	1.54 (1.25-1.90)	0.000
Bilateral liver disease	1.21 (0.99-1.47)	0.065	0.98 (0.75-1.27)	0.864
RAS stasus				
Wild-type tumors	Ref		Ref	
Mutated	1.96 (1.61-2.40)	0.000	1.90 (1.55-2.33)	0.000
BRAF status				
Wild-type tumors	Ref			
Mutated	1.17(0.38-3.65)	0.768		
Extrahepatic disease	1.95 (1.53-2.50)	0.000	2.03 (1.58-2.61)	0.000
Major resection	1.65 (1.32-2.06)	0.000	1.40 (1.11-1.77)	0.004
Red Blood Cell Transfusion	1.40 (0.99-1.97)	0.055	1.03 (0.72-1.48)	0.871
R1 resection	1.24 (0.99-1.56)	0.056	1.25 (0.99-1.58)	0.058
Adjuvant chemotherapy	0.64 (0.52-0.79)	0.000	0.65 (0.53-0.82)	0.000

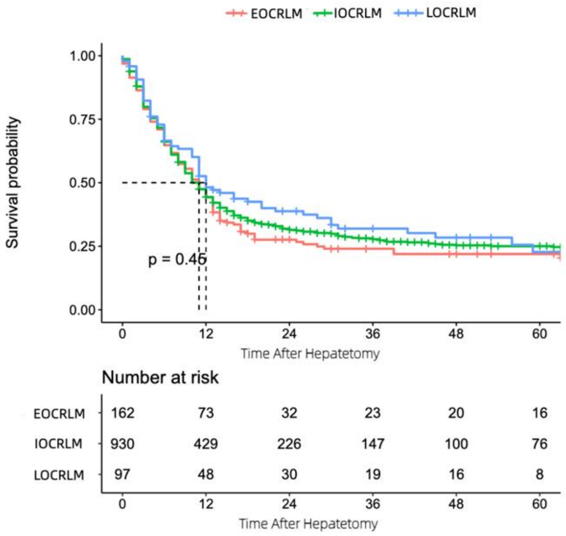

Figure S1. Kaplan–Meier estimates of disease-free survival stratified by age groups in the whole cohort.

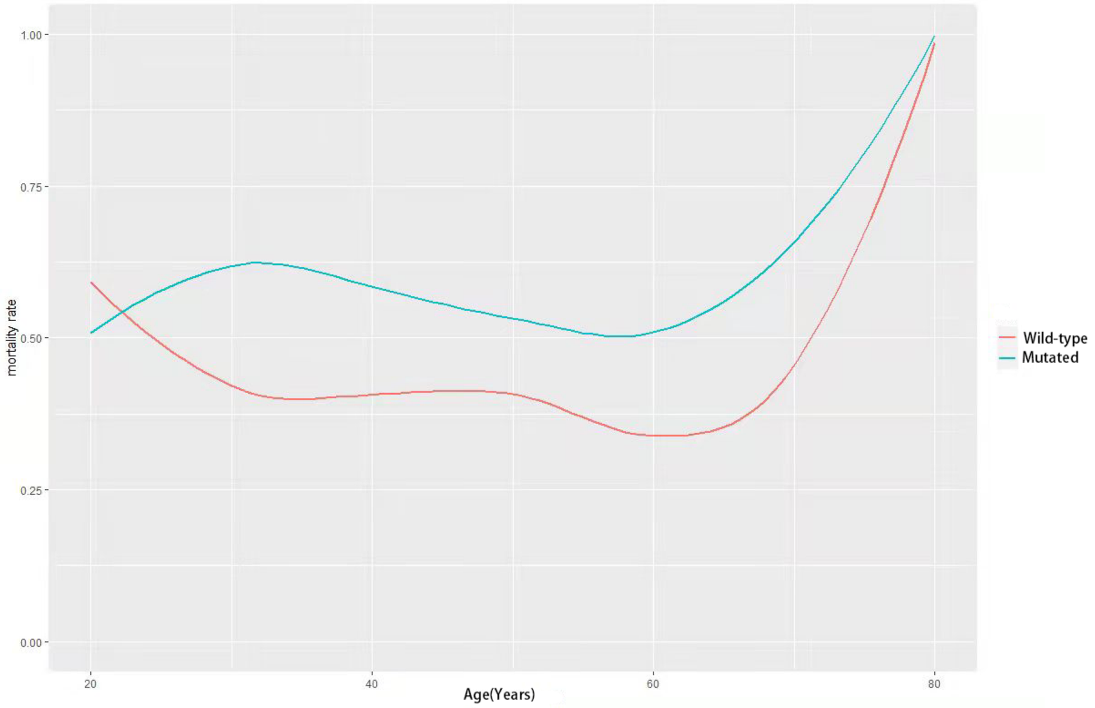

Figure S2. The age-specific mortality risk by RAS status.

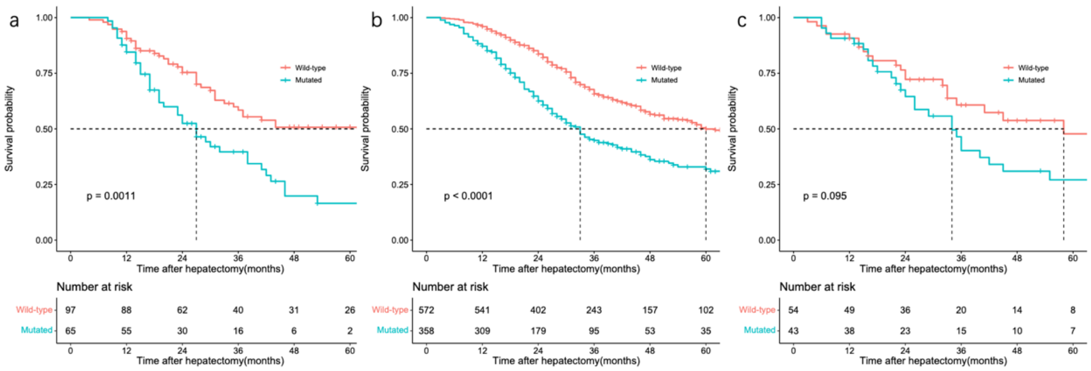

Figure S3. Survival analysis according to the RAS stratification a, b and c: Kaplan–Meier curves of the EOCRLM group patients(a), IOCRLM group(b) and LOCRLM group(c) stratified by the RAS status.
